# Woman and the Bicycle

**Published:** 1896-06

**Authors:** 


### WOMAN AND THE BICYCLE.

SO MUCH has been said and written about the advantages and disadvantages of bicycling, especially for women, that it would be well-nigh impossible to add anything new on the subject. There are now probably very few physicians who would not recommend bicycling as a safe and healthful form of exercise to a woman without grave organic disease. It should, in our estimation, be forbidden where grave disease of the pelvic organs and acute disease of the bladder exists, but in the minor pelvic ailments it might safely be tried ; for the increased amount of oxygen introduced into the system, the swifter circulation and the mental exhilaration would be powerful tonic factors and be especially serviceable in cases associated with hypochondriasis. It is hardly safe to allow persons with organic heart disease to ride, although Tiburties, in a recent number of the *Deutsche Medicinische Wochenschrift*, of April 2d, claims that in his own case bicycling regulates the heart's action, and he rides with impunity, although suffering from atheroma and a moderate amount of emphysema.

That bicycling produces a sense of wellbeing, both physical and mental, that it increases the appetite and promotes sleep, cannot be disputed by anyone who has ever ridden. A woman especially, however, should remember that all the benefits derived from bicycling will be nullified if it is carried to excess. The rides should be judiciously graduated, commencing with a few miles and very gradually increased in length, but never carried to the point of physical exhaustion.

Hills of any size should not be attempted until they can be ascended with comparative ease. Riding should be forbidden during the menstrual periods. The clothing should be light and comfortable, the underwear preferably of wool or silk. The corset should be discarded, as it prevents the full expansion of the lung and impedes the circulation, and as a good substitute can easily be found in the Ferris or equipoise waist.

There should be no constriction about the limbs, as that would impede the circulation and lead to the formation of varicose veins. The objections which have heretofore been raised against faulty saddles have been removed to a great extent by the Messinger and Christy saddles, which appear to meet all the indications.

The saddle should be so adjusted that the extremity is not completely extended as it reaches the lowest point of the pedal, for complete extension favors a tilting forward of the pelvis. The handle-bar should be arranged in such a way that the forearm is slightly flexed.

If the foregoing points are observed and conscientiously carried out by the rider, bicycling is one of the most wholesome and delightful forms of exercise a woman can take. It is especially to be recommended in neurasthenic patients, in those suffering from derangement of the digestive system, particularly when complicated with constipation and in cases where the system is below par, the result of a sedentary mode of life.

